# Green pyomelanin-mediated synthesis of gold nanoparticles: modelling and design, physico-chemical and biological characteristics

**DOI:** 10.1186/s12934-019-1254-2

**Published:** 2019-12-03

**Authors:** Imen Ben Tahar, Patrick Fickers, Andrzej Dziedzic, Dariusz Płoch, Bartosz Skóra, Małgorzata Kus-Liśkiewicz

**Affiliations:** 1 Microbial Processes and Interactions, TERRA Teaching and Research Centre, Gembloux Agro-Bio Tech, University of Liege, Avenue de la Faculté, 2, 5030 Gembloux, Belgium; 2 Institute of Physics, College of Natural Sciences, University of Rzeszow, Pigonia 1, 35-310 Rzeszow, Poland; 3 Department of Biotechnology, Institute of Biology and Biotechnology, College of Natural Sciences, University of Rzeszow, Pigonia 1, 35-310 Rzeszow, Poland

**Keywords:** Pyomelanin, *Yarrowia lipolytica*, Quadratic model, Gold nanoparticle, Cytotoxicity, Mouse fibroblasts, Human osteosarcoma cells

## Abstract

**Background:**

Synthesis of nanoparticles (NPs) and their incorporation in materials are amongst the most studied topics in chemistry, physics and material science. Gold NPs have applications in medicine due to their antibacterial and anticancer activities, in biomedical imaging and diagnostic test. Despite chemical synthesis of NPs are well characterized and controlled, they rely on the utilization of harsh chemical conditions and organic solvent and generate toxic residues. Therefore, greener and more sustainable alternative methods for NPs synthesis have been developed recently. These methods use microorganisms, mainly yeast or yeast cell extract. NPs synthesis with culture supernatants are most of the time the preferred method since it facilitates the purification scheme for the recovery of the NPs. Extraction of NPs, formed within the cells or cell-wall, is laborious, time-consuming and are not cost effective. The bioactivities of NPs, namely antimicrobial and anticancer, are known to be related to NPs shape, size and size distribution.

**Results:**

Herein, we reported on the green synthesis of gold nanoparticles (AuNPs) mediated by pyomelanin purified from the yeast *Yarrowia lipolytica*. A three levels four factorial Box–Behnken Design (BBD) was used to evaluate the influence of temperature, pH, gold salt and pyomelanin concentration on the nanoparticle size distribution. Based on the BBD, a quadratic model was established and was applied to predict the experimental parameters that yield to AuNPs with specific size. The synthesized nanoparticles with median size value of 104 nm were of nanocrystalline structure, mostly polygonal or spherical. They exhibited a high colloidal stability with zeta potential of − 28.96 mV and a moderate polydispersity index of 0.267. The absence of cytotoxicity of the AuNPs was investigated on two mammalian cell lines, namely mouse fibroblasts (NIH3T3) and human osteosarcoma cells (U2OS). Cell viability was only reduced at AuNPs concentration higher than 160 µg/mL. Moreover, they did not affect on the cell morphology.

**Conclusion:**

Our results indicate that different process parameters affect significantly nanoparticles size however with the mathematical model it is possible to define the size of AuNPs. Moreover, this melanin-based gold nanoparticles showed neither cytotoxicity effect nor altered cell morphology.
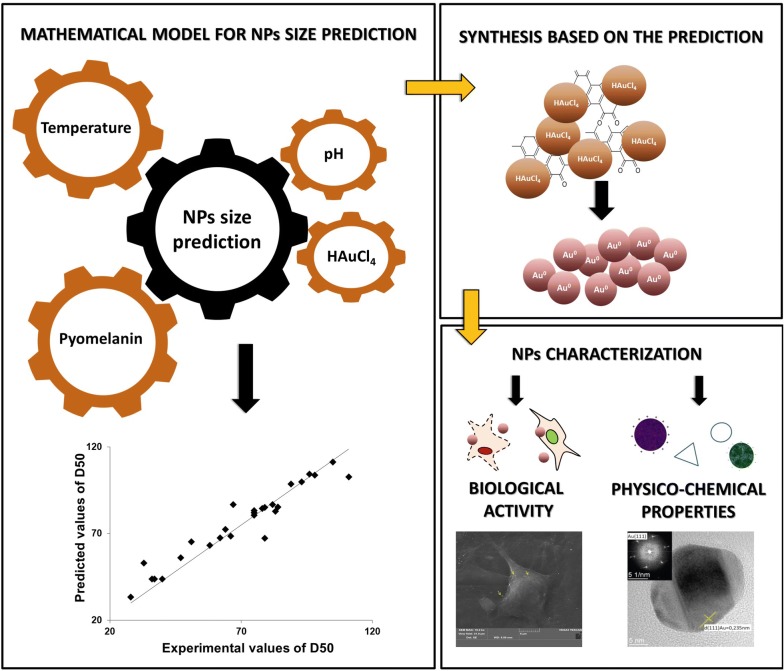

## Background

Nanoparticles (NPs) are an emerging field of nanotechnology research due to their potential applications in medicine [1, 2], cosmetics [3], textile [4], construction [5], renewable energies [6] and many other areas. Several types of metal NPs such as gold, silver, titanium dioxide or copper, have been manufactured and used for different applications in biological and medical fields based on their specific features [7]. Among them, gold nanoparticles (AuNPs) exhibit peculiar physico-chemical proprieties. They are chemically inert and highly stable once synthesized [8]. They present a large surface area, high electron conductivity and uncommon optical properties [9]. Furthermore, the ability of AuNPs to bind ligands by interacting with their amine and thiols groups provides a versatile mean to generate specific biomarkers and conjugating therapeutic agents [10, 11]. Depending on the type and concentration of the ligand bound on the surface these particles; their immunogenic response, reactivity, stability and sensitivity could be modulated [12]. All these benefits, combined with their low cytotoxicity, enable their utilization in different biomedical applications including computing tomography [13, 14], photoacoustic imaging [15], drugs and genes delivery systems [16–18], photothermal therapy [19], radiosensitization [20] and biochemical sensing [21].

Green methods based on the utilization of microbial cells or their metabolites for NPs synthesis has become popular over the years as an alternative to the classical chemical approach [22–25]. Melanins that form a heterogenous group of biopolymers, including eumelanin, pyomelanin, allomelanin, have been used for NPs synthesis [23, 26–28] based on their redox proprieties [29] and affinity for metal ions [30]. Microorganisms, such as the yeast *Yarrowia lipolytica,* have been described for their ability to produce melanin, namely pyomelanin, with high productivity [31]. Despite melanin mediated synthesis of AuNPs is innovative and promising, the control of process parameters (temperature, pH, and concentrations of reactants) is still not efficient enough to modulates the NPs size and shape, and thus their possible application [24]. Therefore, extended knowledge on the interactions between these process parameters is requested. Herein, a mathematical model describing the relation between these process parameters and AuNPs size was build and validated experimentally. The synthesized AuNPs were then characterized and tested for their toxicity on mammalian cell lines. For this purpose, cell metabolic activity, cell viability and morphology analysis were performed after cell exposure to pyomelanin synthesized AuNPs.

## Results and discussion

### Effect of temperature, pH, pyomelanin and gold concentrations on NPs size

Four main parameters, namely temperature, pH, metal salt concentrations and reducing agent concentrations are known to affect strongly NPs size distribution and therefore their properties [24]. Hence, efficient tools to master size distribution during NPs synthesis are required. The traditional approach, one factor at a time, considers only the variation of one parameter while keeping all the others at a constant level [32]. This approach excludes any eventual interactions between parameters on NPs size distribution. Moreover, it requires an important number of experiments that are time consuming. Box–Behnken design (BBD) is a well-known optimization approach used to generate a mathematical model that considers the interactions between parameters on the final output (i.e. particle size). It offers high prediction ability and requests only a low number of experiments to be set-up. Herein, a three levels four factorial BBD, was used to define the influence of temperature, pH, gold salt and pyomelanin concentrations on nanoparticles size distribution. According to the BBD design, 27 experimental conditions were considered. The NPs size distribution (PSD, polydisperse size distribution) for each experiment was analyzed by dynamic light scattering (DLS) and the median value of the size distribution (D50) was used as output response.

In the BBD, the upper and lower levels of pH, and gold salt concentrations were fixed on the basis of previous studies [33, 34]. Unfortunately, the effects of temperature and pyomelanin concentration on AuNPs size are still unknown. Therefore, these values were defined in an extended range of experimental region (10–90 °C and 500–1000 g/mL, respectively). The PSD for the 27 experimental conditions are presented in Additional file 1: Table S1. Results highlighted a strong fluctuation of D50 values with the level (i.e. low, medium, high) of each parameter. They ranged between 28 and 111 nm, highlighting the key role of the studied parameters on the AuNPs size distribution. Based on the BBD design and D50 values, a quadratic model as well as the model validation with graphical residuals were established (for details see Additional file 1).

**Fig. 1:**
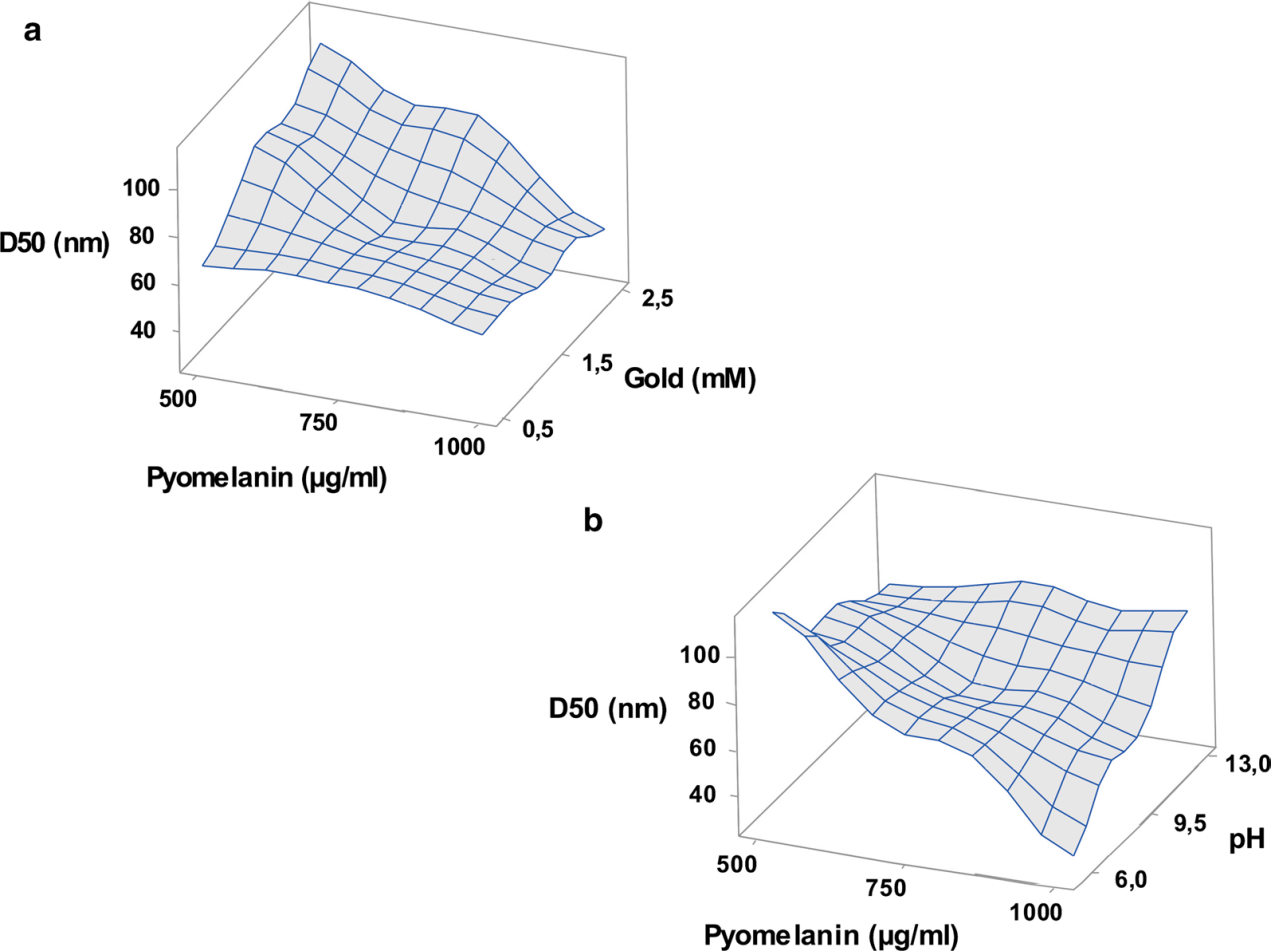
Three-dimensional response surface curve for AuNPs synthesis highlighting the interactive effects of **a** gold and pyomelanin concentrations; **b** pyomelanin concentration and pH

In order to better analyze the interactions between the different parameters on D50 values, two-dimensional response surface plots were drawn using the established model. On each plot, D50 values were on Z-axis while X and Y-axis were assigned for two of the significant parameters. The remaining variables were kept at their medium level. As shown in Fig. 1a, the highest D50 values were obtained at high salt concentration and low pyomelanin concentration. By contrast, lower D50 values were obtained when gold salt and pyomelanin concentrations were fixed at their low and high levels, respectively. Beside this, the highest D50 values were obtained at high pH and pyomelanin concentrations (Fig. 1b). On the other hand, lower pH and high pyomelanin concentration reduced remarkably the AuNPs size and the smallest D50 values were obtained in these conditions. In the literature, reports on the assessment the effects of process parameters on the synthesis of pyomelanin-mediated nanoparticles are scare. Here, a surface response methodology was used to establish a mathematical model in order to highlight the influence of different physico-chemical parameters and their interaction on the AuNPs PSD value. Previous reports focused on the influence of various physico-chemical parameters using one factor at a time methodology. Apte et al. reported a significant variation of UV–Visible spectra of AuNPs when the concentration of gold salt and melanin varied separately, suggesting that these factors affect the nanoparticles PSD [35]. In the same context, the effect of pH and gold salt concentration on AuNPs synthesis was reported [33]. At a fixed concentration of melanin, the UV–visible spectrum of AuNPs is remarkably varying according pH or salt concentration.

### Physico-chemical characterization of AuNPs

For medical applications of AuNPs such as imaging or drug delivery system, particle size should be in the range of 10 nm and 150 nm [36, 37]. Therefore, to synthesize such AuNPs, experimental conditions were defined using the developed model, namely a melanin concentration of 500 µg/mL, pH 6 and HAuCl_4_ concentration of 1.5 mM. Reduction of metal ions was initially monitored by visual observation, and change of color. After 24 h of incubation of the reaction mixture at 50 °C, a purple color appeared, demonstrating the formation of gold nanoparticle [9, 38]. By contrast, control samples (pure pyomelanin and HAuCl_4_) showed no color change when incubated under the same conditions.

**Fig. 2:**
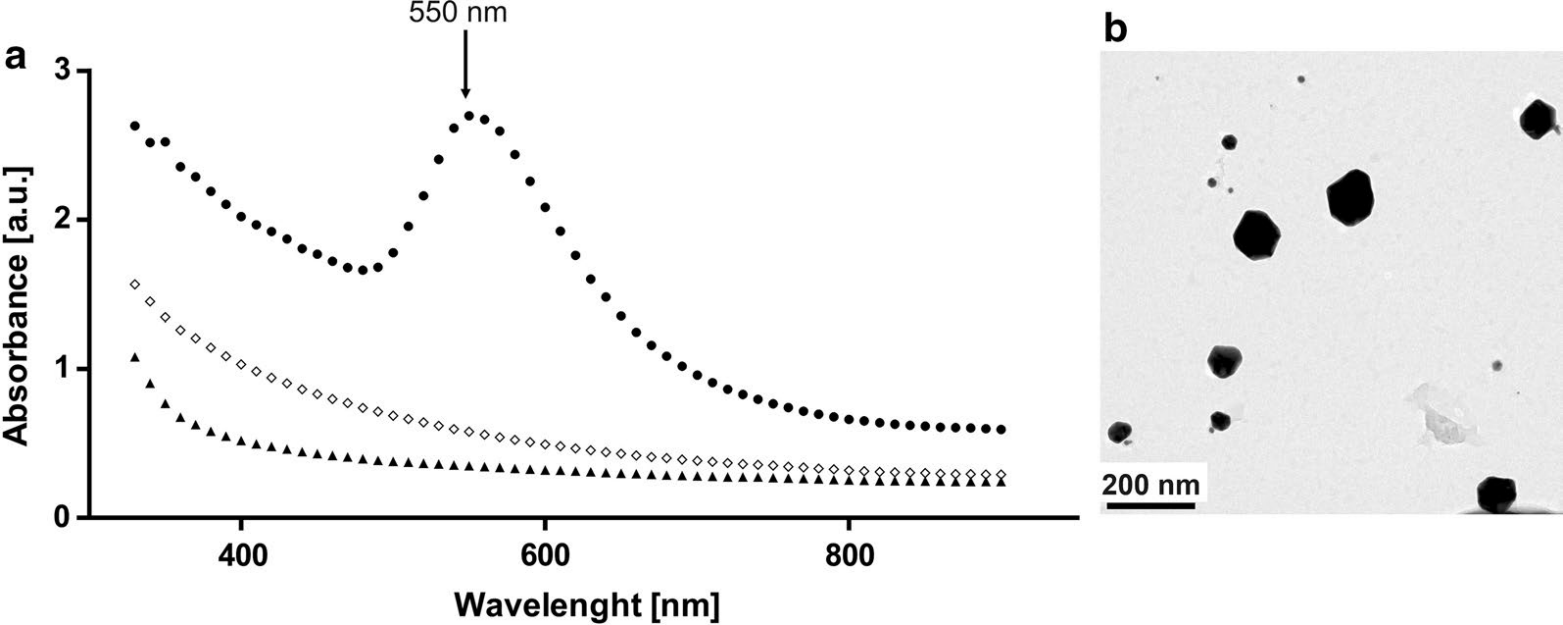
**a** UV–Vis absorption spectra of AuNPs synthesized from melanin (circle), pure pigment (diamond) and HAuCl_4_ (triangle). Plasmon peak (with wavelength value) was shown. **b** TEM image of the AuNPs

As a first characterization of the synthetized AuNPs, the UV–Visible absorbance spectrum was recorded together with that of pure pyomelanin and HAuCl_4_ (Fig. 2a). For synthetized NPs, the absorbance peak observed at 550 nm is due to an effect of local surface plasmon resonance (LSPR) phenomenon [39]. Indeed, it has been demonstrated that LSPR depends on the size, shape and type of metal nanoparticles. For small AuNPs (between 5 and 10 nm), the LSPR band appears around 520 nm, while for bigger particles (between 50 and 100 nm), this peak is red shifted up to 570 nm [9, 40]. Furthermore, in the UV–Vis spectrum, one single absorbance peak was recorded, suggesting the spherical shape of the particle. Indeed, when the nanoparticles have an anisotropic shape, two or more plasmon bands occur as a result of electrons oscillation along two or more axes. By contrast, in the case of spherical NPs, a single peak is detected [41]. To confirm this hypothesis AuNPs were analyzed by transmission electron microscopy (TEM). As shown in Fig. 2b, the particles are of spherical shape, confirming thus result from UV–Vis spectrum. The NPs size distribution was assessed by DLS, which measure the hydrodynamic diameter of particles in a suspension [42]. The synthetized particles presented a size with median value 104 nm (± 5 nm).

For many applications, NP stability (i.e. the lack of particle aggregation) is a prerequisite as particle size affects the biological activity or their cytotoxicity [43]. Therefore, Polidispersity Index (PDI) and Zeta potential (ζ) values were estimated for the synthetized AuNPs. PDI shows the ratio of particles of different size to total number of particles. The higher the PDI value is, the less monodispersed are the nanoparticles [39]. In this study, PDI values were found equal to, respectively, 0.276 and 0.252 upon synthesis and after 3 months of storage at 4 °C. These data are very close to the typical and commonly deemed value (~ 0.2) acceptable in practice [44]. The Zeta potential values were also determined over time for the AuNPs. The corresponding ζ values were − 28.96 mV and − 31.80 mV, respectively. This highlighted the stability of the particle colloid [45]. Zeta potential measurement may provide also an insight on the nature of the interaction between the cells and nanoparticles. Indeed, the surface charge of the particle influences significantly the cellular uptake of the NPs. It is commonly admitted that positively charged NPs are more internalized by cells than neutral or negatively charged one. As a consequence, this higher intracellular accumulation leads to a faster destruction of the cell integrity, leading thus to a higher cellular toxicity. By contrast, anionic or neutral NPs have a lower affinity for cell since this latter has an over whole negative charge. Consequently, the membrane depolarization after exposure to the ^−^AuNPs or ^0^AuNPs is negligible as compared to positively charged one [46–48]. This could explain the lack of toxicity of the pyomelanin- synthesized AuNPs that are negatively charge particle. This characteristic is mandatory for any nanoparticles that are being considered for biomedical application [49].

**Fig. 3:**
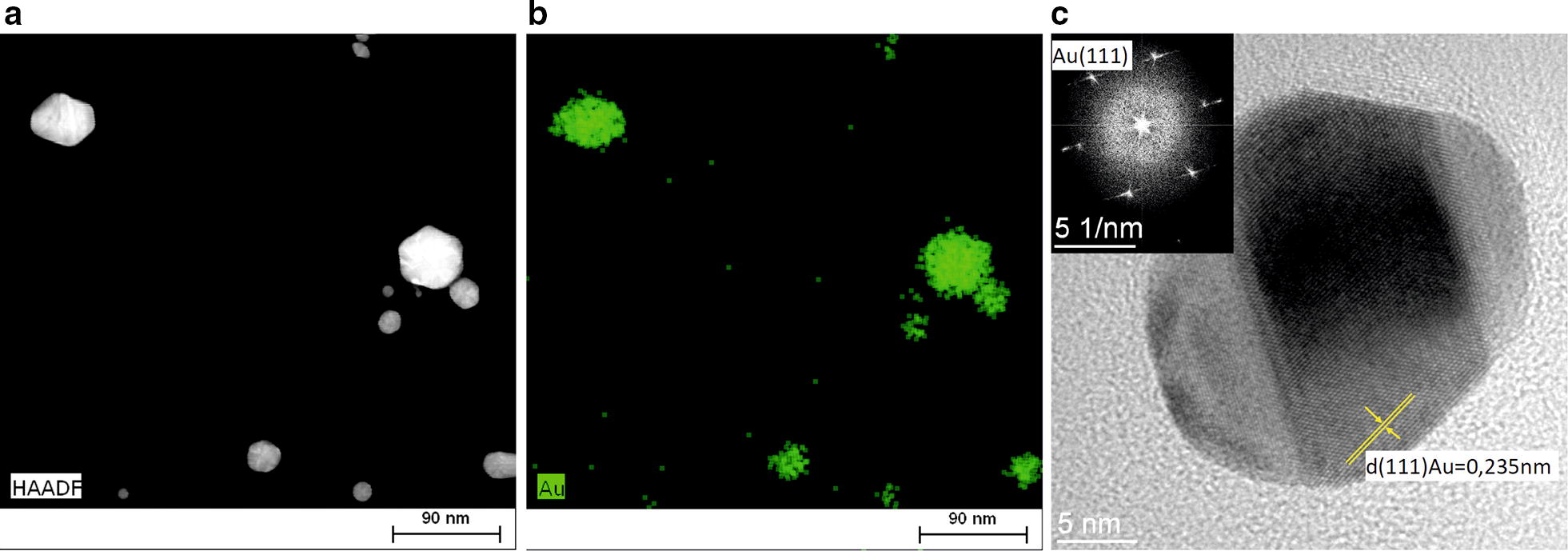
**a** HAADF STEM image of AuNPs shows the spherical or polygonal nanoparticles. **b** The chemical composition analysis of AuNPs. EDS mapping of the Au (green) localized the gold nanoparticles. **c** Representative HRTEM image of AuNPs. Gold was identified by the inter-planes spacing d = 0.235 nm corresponding to the (111) plane of gold. Measurement of inter-planes spacing was done on FFT image (**c**, inset)

As a further characterization, the AuNPs were analyzed by TEM and energy dispersive X-ray spectroscopy (EDS). According to the high-angle annular dark-field in a scanning transmission electron microscope (HAADF STEM) photographs, the AuNPs displayed either a polygonal or a spherical shape (Fig. 3a). EDS was used to characterize the elemental compositions of synthesized particles. In this analysis, the atoms on the NPs are excited by the electron beam of specific wavelength. These in turn emit X-rays at energy that is element specific [50]. As shown in Fig. 3b, the green EDS mapping highlights that the particle are composed of gold since the green color is centered on the particle. The crystallographic structure of AuNPs was deduced from high-resolution transmission electron microscopy (HRTEM) images processed by Fast Fourier Transformation (FFT). As shown in Fig. 3c, the AuNPs are of crystalline structure consisting of several crystalline facets with well-defined inter-planer spacing of 0.235 nm, corresponding to the (111) gold plane [51].

### In vitro biological study

Although, the synthesis of AuNPs using melanin as reductant has been reported, there is no data of the characterization of their cytotoxicity on eukaryotic cells [52]. Herein, this characterization was performed through different but complementary methods. For that purpose, two mammalian cell lines were used, namely mouse fibroblasts (NIH3T3) and human osteosarcoma cells (U2OS).

**Fig. 4:**
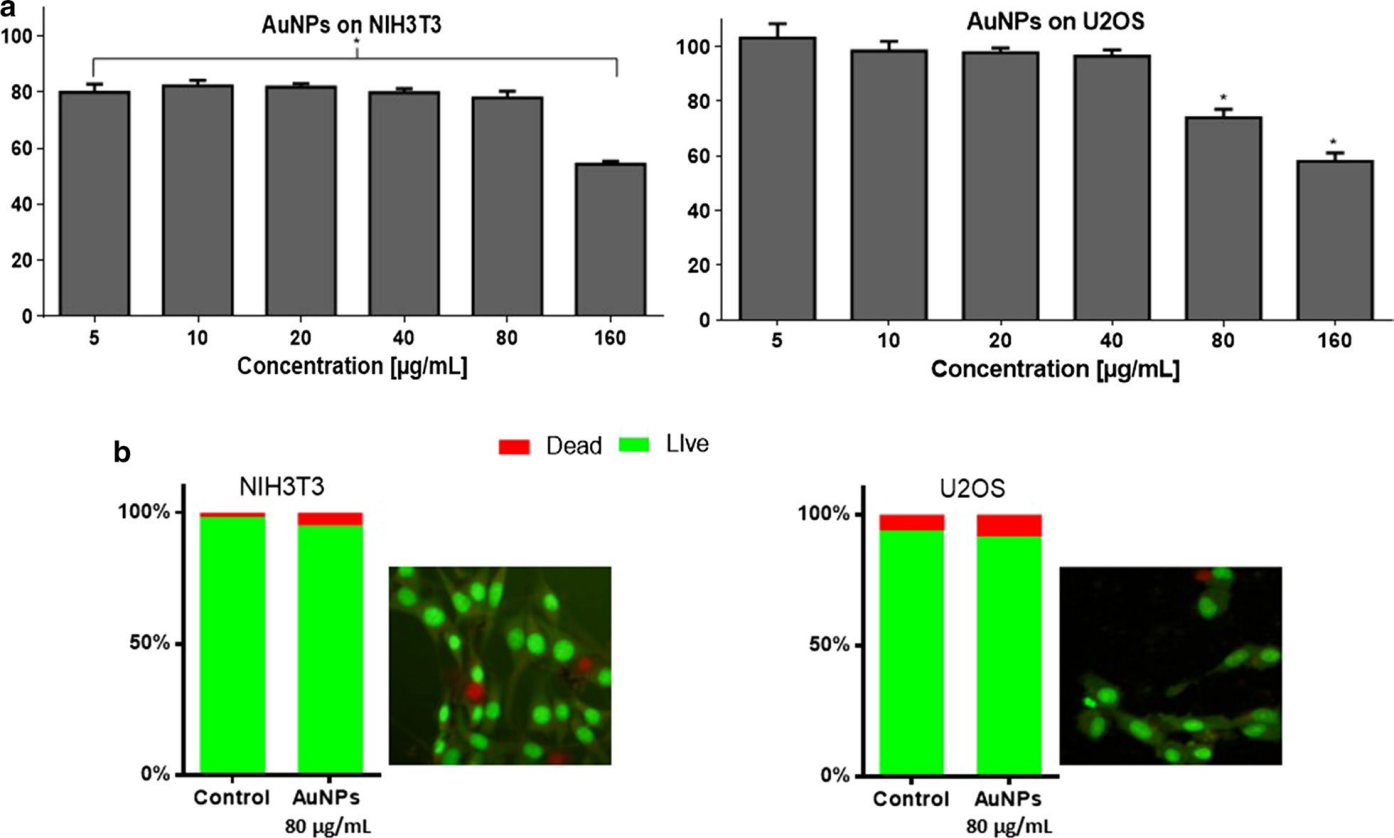
Cell metabolic activity; MTT assay (**a**) and cell viability; dual staining assay (**b**) of mouse fibroblasts (NIH3T3) and human osteosarcoma (U2OS) after exposure to melanin-based gold nanoparticles. For MTT assay, cells were incubated for 24 h with different amounts of AuNPs. Results are presented as a percentage of metabolically active cells after AuNPs melanin treatment (three replicates with ± SD) compare to control (without AuNPs). Statistical analysis was performed using one-way ANOVA and Dunett’s a posteriori. *p < 0.05, no asterisk indication—no statistical significance. For dual staining, cells after AuNPs treatment were stained with acridine orange and ethidium bromide. Cells were imaged with inverted fluorescence microscope, objective ×20. Dead cells were scored per 100 total cells analyzed and expressed as %

The MTT assay consists to monitor any reduction in the ability of cells to degrade MTT into formazans in the presence of a potential perturbator (i.e. AuNPs). For these tests, confluent NIH3T3 and U2OS cells were incubated in the presence of different concentrations of AuNPs (5–160 µg/mL). After 24 h of incubation, the cell metabolic activity was assessed based on the conversion of MTT into formazans that were quantified by spectrophotometry. In the range 5–80 µg/mL of AuNPs, both cell lines remained metabolically active (Fig. 4a). For the concentration of 160 µg/mL, U2OS and NIH3T3 cell metabolic activity was reduced by 42% and 46%, respectively, suggesting thus, their cytotoxicity at that concentration.

Cell viability was also investigated by dual staining with acridine orange and ethidium bromide. For this purpose confluent cells were incubated for 24 h with AuNPs at a concentration of 80 µg/mL (the highest concentration with no significant cytotoxicity). As shown in Fig. 4b, the cellular mortality was, respectively, 9% and 5% for U2OS and NIH3T3. This confirmed the lack of cytotoxicity of AuNPs at concentration lower than 80 µg/mL.

**Fig. 5:**
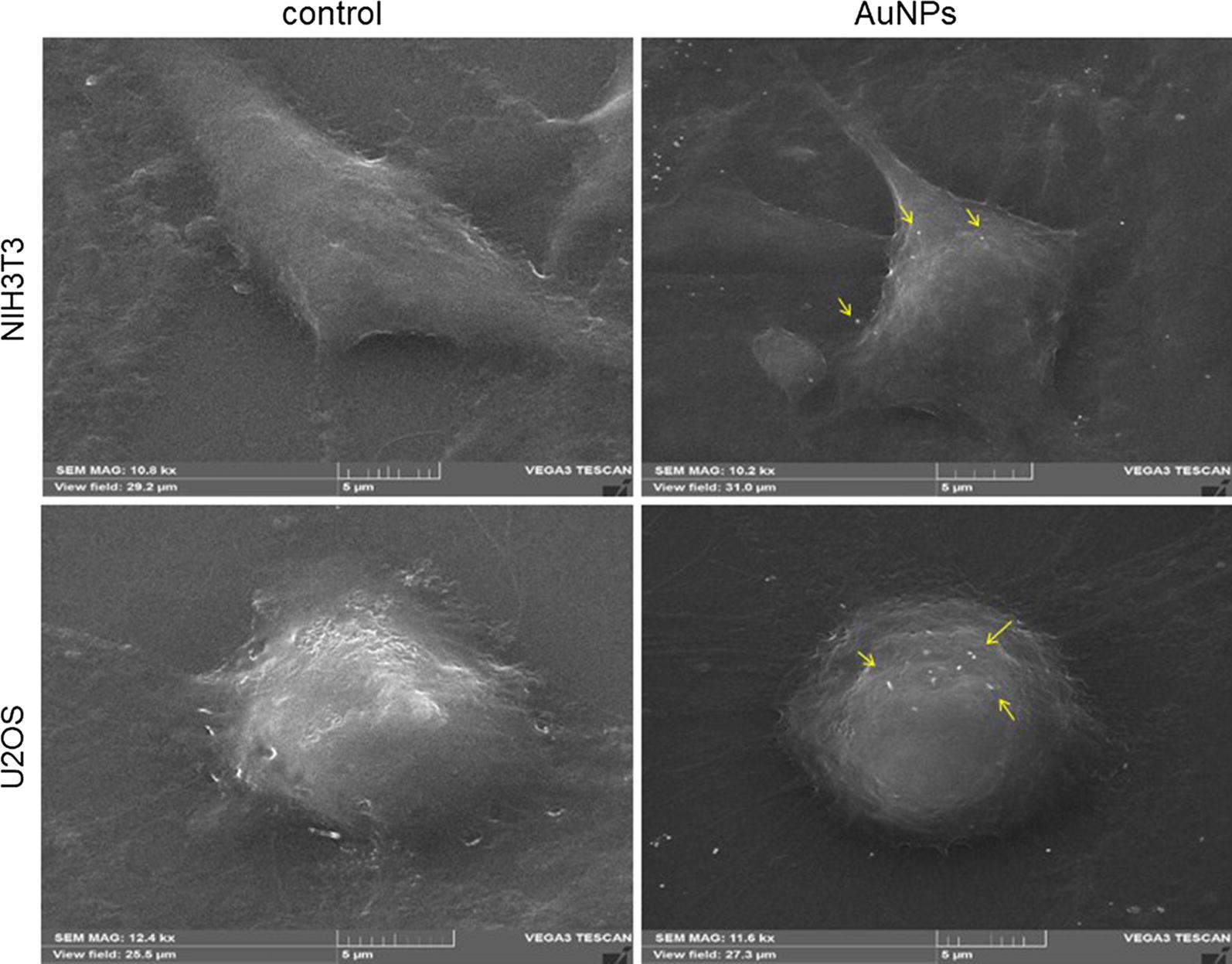
SEM micrographs representing the morphology of the mouse fibroblasts (upper) and human osteosarcoma (lower) cells without (control) or upon contact with AuNPs. The gold nanoparticles were deposited on the cell surface (yellow arrows). Scale bars 6 µm. Operating parameters: det SE, HV = 20–30 kV, Mag = 10–12 kx, Specimen Current = 30–100 pA

Since cells morphology depends on the surrounding environment [53], the effect of AuNPs on cells shape and ultrastructure were examined with SEM. Despite particles were found non-cytotoxic, they could have an impact on cell adherence ability and morphology. Therefore, confluent U2OS and NIH3T3 cells were incubated with AuNPs (80 µg/mL) for 24 h before being visualized by SEM. After incubation, the cells were still seen as covering the surface homogenously regardless the AuNPs treatment (data not shown). As shown by SEM imaging, for a both cell lines, incubation with AuNPs did not modify the cell morphology (representative images are shown in Fig. 5). NIH3T3 exhibited elongated fibroblast-like shape, either in treated or non-treated cells. For U2OS, both, the control and AuNPs-treated cells, displayed similar morphology; they have a less flattened and more rounded shapes. Moreover, the cells exhibited protruding extensions making cell-surface focal contacts. Such structure, these filaments barbed-ends, is formed with the cytoskeleton proteins, and is pivotal for cell motility, membrane domains organization as well as for cell growth and proliferation [54, 55]. Thus, this points out that AuNPs had no negative effect on the morphology of the tested cells. SEM images showed also that gold nanoparticles were bound to the cell surface (Fig. 5, arrows). These boundaries could results from the protein corona that could be form by interaction of the protein with surface of the NPs [56]. This phenomenon is an important issue when designing and manufacturing NPs for cellular uptake or biofunctionalization. Indeed, proteins or peptides released from cells could adsorbed on the surface of AuNPs and influencing, thus, their nanotoxicity [57]. Here, the lack of the cytotoxicity of AuNPs can be explained by the protein corona formed around the NPs which acts as a protective layer [58]. Protein corona being formed upon incubation of the AuNPs with serum from cell culture medium. Protein corona may also has an impact on the NPs stability. Generally, nanoparticles are unstable due to their high surface energy and must be stabilized by surface modification. Recently, a number of functional groups and peptides were designed for successful gold NPs stabilization (review in [59]). According to our SEM analysis, no evidence of AuNPs agglomeration was noticed, what is convergent with the result obtained with Zeta potential measurement and assessment of the colloid stability.

Such experiments, related to the biological characterization of pyomelanin based gold nanoparticles synthesis have not been reported in the literature so far. We reported a possible manufacturing methodology for stable non-cytotoxic “nanoplatform”, with various biomedical applications.

## Conclusion

Recently, green chemistry methods involving the use of microorganisms or their metabolites, for the synthesis of nanoparticles become a research topic of great interest and turned as an eco-friendly alternative to the conventional chemical approach. Among different green approaches, the synthesis of gold nanoparticles using pyomelanin produced by *Y. lipolytica* is reported here. In the first part of this work, a three levels four factorial Box–Behnken Design, was used to evaluate the influence of temperature, pH, gold salt and pyomelanin concentration on nanoparticle size distribution. Based on statistical analysis, it has been shown that different process parameters affect significantly nanoparticles size. The mathematical model developed was used to predict and manufacture AuNPs with specific size distribution, since this parameter influences greatly their biological properties. The synthesized nanomaterial was characterized by different analytical methods. The NPs were found to have crystalline structure, polygonal and spherical shape, with a DC50 value of 104 nm. In addition, the synthesized nanostructures revealed high colloidal stability and moderate polydispersity. It was also pointed out with SEM analysis, that AuNPs were administered as a well dispersed single particle suspension. Melanin-based gold nanoparticles did not show cytotoxicity effect toward human osteosarcoma and mouse fibroblasts. Moreover, this material did not cause a perturbation in cell morphology after exposure to the AuNPs. Furthermore, our work revealed that brown pigment, pyomelanin, isolated from *Yarrowia lipolytica*, could have a reduction role during NPs synthesis. In summary, our results highlighted the potential of synthesized AuNPs to be used as a powerful tools for bioapplication.

## Materials and methods

### Chemicals, cell lines, yeast strain and culture conditions

Chloroauric acid (HAuCl_4_), 3-(4,5-dimethylthiazol-2-yl)-2,5-diphenyltetrazolium bromide thiazolyl blue tetrazolium bromide (MTT), acridine orange (AO), glutaraldehyde were obtained from Sigma-Aldrich. Dimethyl sulfoxide (DMSO) and ethidium bromide (EtBr) were purchased from Chempur and MpBio, respectively. For cell culture, Dulbecco’s Modified Eagle Medium High Glucose (DMEM, Corning), antibiotic solution (Antibiotic Antimycotic Solution, Sigma-Aldrich), fetal bovine serum (FBS, Biowest), phosphate buffered saline w/o magnesium and calcium (PBS, Corning), 0.25% trypsin (PAA) were supplied from Sigma-Aldrich. The wild strain *Y. lipolytica* W29 (CLIB89) was used for pyomelanin production. Standard techniques used for *Y. lipolytica* have been described elsewhere [60]. Yeast cells were grown in 250 mL conical flask containing 50 mL of YNBTy medium (1.7 g/L yeast nitrogen base (YNB), 1 g/L tyrosine, 1 g/L asparagine, 1 g/L glycine, 10 g/L glucose, 0.15 g/L MnSO_4_). The cell lines used in this study were U2OS (human osteosarcoma) and NIH3T3 (mouse fibroblasts). They were grown and handled according to standard technique as described elsewhere [61]. Cultures were performed in 24- or 96-well plate (Nest) containing 1.5 mL and 200 µL of DMEM medium, respectively, supplemented with 1% of antibiotics solution and 10% of fetal bovine serum (here after stated as DMEM medium) at 37 °C in a 5% CO_2_ air saturated incubators. Cultures were seeded with 5 × 10^5^ and 3 × 10^4^ cells/well for 24- or 96-well plate, respectively.

### Pyomelanin production and purification

*Yarrowia lipolytica* was grown in YNBTy medium for 120 h at 30 °C, on a rotary shaker (150 rpm). Then after, pyomelanin was purified from the culture supernatant as previously described [35]. The culture broth was centrifuged at 10,000×*g* for 15 min and cells were discarded. The supernatant was acidified to a pH 2 with 1 N HCl and incubated at 25 °C for 48 h. After precipitation, pyomelanin was washed once with chloroform and methanol before being dried under vacuum. Then, pyomelanin was solubilized in water alkalinized at pH 8 with 1 N NaOH.

### Response surface methodology

In this study, the effect of temperature, pH, chloroauric acid (HAuCl_4_) and pyomelanin concentrations on gold nanoparticles size has been assessed using Box–Behnken design (BBD) model. The number of experiments is calculated on the basis of the following Eq. (1) [62]:

1 $${\text{N}} = 2{\text{k}}\left( {{\text{k}} - 1} \right) + {\text{C}}_{0}$$

where k is number of factors and C_0_ is the number of central points. In this study, a total of 27 experiments were carried out for a four factors, three levels design with three replicates of the central points. The variable input parameters were temperature, pH, pyomelanin concentration and HAuCl_4_ concentration. The range and levels of pH (6–9.5–13), and gold salt concentrations (0.5–1.5–2.5 mM) were selected on the basis of previous studies [34, 35]. For temperature and pyomelanin concentrations, the lower, medium and upper levels for these parameters were defined in a large range of experimental region, (10–50–90 °C and 500–750–1000 µg/mL, respectively) as these level are unknown so far. The NPs size distribution for each experiment was analyzed by DLS (see below) and the calculated median value of the size distribution (D50) was considered as output response. The general quadratic form of the mathematical model formed by BBD is defined as follow (2) according to [63]:

2 $$\begin{aligned} S &= a_{0} + a_{1} F_{1} + a_{2} F_{2} + a_{3} F_{3 } + a_{4} F_{4} + a_{11} F_{1}^{2} \hfill \\ & \quad + a_{22} F_{2}^{2} + a_{33} F_{3}^{2} + a_{44} F_{4}^{2} + a_{12} F_{1} F_{2} \hfill \\ & \quad + a_{13} F_{1} F_{3} + a_{14} F_{1} F_{4} + a_{23} F_{2} F_{3} \hfill \\ & \quad + a_{24} F_{2} F_{4} + a_{34} F_{3} F_{4} \hfill \\ \end{aligned}$$

where S is the predicted value of D50, a_0_ is the constant, F_1_ F_2_ F_3_ and F_4_ represent temperature, pH, HAuCl_4_ and pyomelanin concentrations, respectively. a_1_,a_2_,a_3_ and a_4_ are linear coefficients, a_12_, a_13_, a_14_, a_23_, a_24_ and a_34_ are cross-product coefficients whereas a_11_, a_22_, a_33_ and a_44_ are quadratic coefficients.

The different coefficients were calculated using the least square method. In order to assess the established quadratic model, statistical analysis of variance (ANOVA) was performed using Minitab 17 (Minitab, USA). The analysis of variance includes the determination of the sum of squares; the mean of squares; Fisher F values and probability *p* values. Moreover, the correlation coefficient R^2^, is used as a mean to assess the fitness of the polynomial model [64]. For the final step of the model validation, a graphical residuals analysis was performed. Residuals are defined as the difference between experimental response and calculated response from the model. The graphical analysis was based on plots depicting the normal probability of residuals distribution, residuals versus the predicted plot and residuals versus the experiment number [65]. The validated model was then used to generate a 3-D graphical representations that depict the influence of parameters on the response (i.e. particle size).

### Characterization of AuNPs

UV–Vis absorbance spectrum of AuNPs, pyomelanin and HAuCl_4_ were recorded on a TECAN spectrofluorometer (Infinite M200, Thermo Scientific) in the range of 330 nm and 900 nm. The hydrodynamic size of AuNPs was measured by DLS, whereas zeta potential was determined by means of electrophoretic light scattering (ELS) using the universal Nanoplus HD3 system (Particulate System/Micrometrics) equipped with 660 nm laser diode, as described elsewhere [66]. For this purpose disposable capillary cells and quartz microcuvette (Particulate System, Small Volume Size Cell) were used, respectively. All the analysis were performed at 25 °C. Prior measurement, solutions of AuNPs were sonicated (310 W, 50 Hz, 100%, 10 min, Polsonic, SONIC-3). For electron microscopy, AuNPs were deposited on a carbon covered copper microscope grid and was left overnight at room temperature until complete drying. They were imaged using TEM (FEI Tecnai Osiris) operating at 200 kV, equipped with EDS detector. To determine the concentration of AuNPs, the microbalance technique using Radwag MYA 5.4Y balance was used. 100 µL of the colloid suspension was placing on aluminium crucible with known mass and evaporating the solvent to a dry mass. Concentration values are given as mean and standard deviation of triplicate. Mammalian cells were visualized on an Olympus BX61 inverted fluorescence microscope equipped with DP72 CCD camera and Olympus CellF software was used for image processing.

### Cytotoxicity of AuNPs

#### Cell metabolic activity and cell viability

To determine the cytotoxicity of AuNPs colloid, two different assays, namely MTT and acridine orange-ethidium bromide staining (AO/EtBr) were employed as described elsewhere [67, 68]. Briefly for MTT assay, confluent U2OS and NIH3T3 cells were first washed with PBS. Two hundreds µL of DMEM medium containing different amounts of AuNPs were then added per culture well. After 24 h of incubation, the 96-well culture plate was centrifuged (300×*g*, 5 min) and the medium was discarded. Two hundreds µL of DMEM medium supplemented with MTT (0.5 mg/mL) were then added per well. After an additional incubation of 2 to 4 h to allow the formazans to form, 100 µL DMSO were added per well to stop the reaction and dissolve the formazans. The absorbance was finally determined at a test wavelength of 570 nm and a reference wavelength of 630 nm. Cell viability was calculated according to Eq. (3) and expressed as a percentage relative to that of the non-treated cells. All tests were performed at least in triplicate.

3 $$Cell\;viability = \frac{{\left( {A570 - A630} \right)treated}}{{\left( {A570 - A630} \right)non{\text{-}}treated}} \times 100$$

For AO/EtBr double straining, confluent U2OS and NIH3T3 cells incubated for 24 h in the presence of 80 μg/mL of AuNPS were centrifuged (300×*g*, 5 min), and the supernatant was discarded. Cells were then washed once with PBS before being stained with a solution of acridine orange (100 μg/mL in PBS) and ethidium bromide (100 μg/mL in PBS) at a volume ratio of 1:1 for 5 min. Under fluorescence microscope, living cells were visualized as green while dead cell were stained in red. For cell viability determination, a total of 100 cells from each cultures were counted and dead cells were expressed as a percentage of the total number of the cells [68].

#### SEM imaging

U2OS cells were seeded (5 × 10^5^ cells/well) onto the glass discs (Menzel Glaser) inserted on 24-well microplate (Nest) and were allow to grow until they reached the confluence. Then, the culture medium was replaced by fresh DMEM supplemented with AuNPs (80 µg/mL). After 24 h of incubation, the cell morphology was observed by Scanning Electron Microscope (SEM, Tescan VEGA 3). For this purpose, medium was discarded and cells attached onto the glass discs were washed twice with PBS. The cell monolayer was then fixed with 2,5% glutaraldehyde for 24 h at 4 °C before being dehydrated using a graded ethanol concentrations from 30 to 100%. Prior to SEM observation, the samples were sputter-coated with a 3 nm gold layer to make them electronically conductive and to avoid electronic charging during SEM imaging. SEM analysis were performed using a TLD electron detector with the following operating parameters: voltage 20–30 kV, specimen current 30–100 pA and magnification 10–12 kx.

## Supplementary information

**Additional file 1.** Additional tables and figures.

## Data Availability

The datasets used and/or analyzed during the current study are available from the corresponding author on reasonable request.
