# Supplementary material for: Green pyomelanin-mediated synthesis of gold nanoparticles: modelling and design, physico-chemical and biological characteristics
Source: Microb Cell Fact. 2019 Dec 3;18:210. doi: 10.1186/s12934-019-1254-2 (PMC6891958; doi:10.1186/s12934-019-1254-2)
Supplement: Supplementary file 1 — Additional file 1. Additional tables and figures. [file 12934_2019_1254_MOESM1_ESM.docx]

Additional material

Green pyomelanin-mediated synthesis of gold nanoparticles: modelling and design, physico-chemical and biological characteristics

Imen Ben Tahar^1^, Patrick Fickers^1^, Andrzej Dziedzic^2^, Dariusz Płoch^2^, Bartosz Skóra^3^, Małgorzata Kus-Liśkiewicz^3,^ *

^1^Microbial Processes and Interactions, TERRA Teaching and Research Centre, University of Liège - Gembloux AgroBio Tech, Avenue de la Faculté, 2. B-5030 Gembloux, Belgium

^2^Institute of Physics, College of Natural Sciences, University of Rzeszow, Pigonia 1, 35-310 Rzeszow, Poland

^3^Department of Biotechnology, Institute of Biology and Biotechnology, College of Natural Sciences, University of Rzeszow, Pigonia 1, 35-310 Rzeszow, Poland

*Corresponding author: Małgorzata Kus-Liśkiewicz, mkus@ur.edu.pl

Table S1. Experimental D50 values under different physico-chemical conditions

| Run | Melanin (µg/ml) | Gold (mM) | Temperature (°C) | pH | D50 (nm) |
| --- | --- | --- | --- | --- | --- |
| 1 | 500 | 1.5 | 50 | 13 | 75 |
| 2 | 750 | 1.5 | 90 | 13 | 98 |
| 3 | 1000 | 1.5 | 10 | 9.5 | 33 |
| 4 | 750 | 0.5 | 50 | 6 | 51 |
| 5 | 750 | 0.5 | 10 | 9.5 | 79 |
| 6 | 500 | 1.5 | 50 | 6 | 113 |
| 7 | 500 | 1.5 | 90 | 9.5 | 111 |
| 8 | 750 | 2.5 | 90 | 9.5 | 96 |
| 9 | 1000 | 1.5 | 50 | 13 | 84 |
| 10 | 500 | 0.5 | 50 | 9.5 | 66 |
| 11 | 1000 | 1.5 | 50 | 6 | 28 |
| 12 | 1000 | 2.5 | 50 | 9.5 | 47 |
| 13 | 750 | 2.5 | 50 | 6 | 83 |
| 14 | 750 | 1.5 | 50 | 9.5 | 37 |
| 15 | 750 | 1.5 | 90 | 6 | 82 |
| 16 | 750 | 1.5 | 50 | 9.5 | 36 |
| 17 | 500 | 1.5 | 10 | 9.5 | 75 |
| 18 | 750 | 1.5 | 50 | 9.5 | 40 |
| 19 | 750 | 1.5 | 10 | 13 | 78 |
| 20 | 750 | 0.5 | 90 | 9.5 | 67 |
| 21 | 1000 | 1.5 | 90 | 9.5 | 64 |
| 22 | 750 | 0.5 | 50 | 13 | 75 |
| 23 | 750 | 2.5 | 50 | 13 | 93 |
| 24 | 500 | 2.5 | 50 | 9.5 | 105 |
| 25 | 750 | 1.5 | 10 | 6 | 62 |
| 26 | 1000 | 0.5 | 50 | 9.5 | 58 |
| 27 | 750 | 2.5 | 10 | 9.5 | 79 |

***Mathematical model***

Based on the BBD design and D50 values, a quadratic model was established and an analysis of variance (ANOVA) was performed using Minitab 17. The significance of each coefficient was evaluated on the basis of its *p-*value. By neglecting the insignificant coefficients (*p*-value>0.05), the mathematical relationship between the output, namely D50, and the input variables, namely temperature, pH, salt gold and pyomelanin concentrations is given by the following equation (1):

D50 = 364 - 0.175A- 24B - 1.073C - 43.56D + 20.181B^2^ + 0.00910C^2^ + 1.627D^2^ - 0.05AB + 0.02AD + 0.18BC

$D50=450.8-0.45A+12B-1.17C-42.41D+{0.000183A^{2}+ 19.46B}^{2}+{0.01411C}^{2}+{1.57D}^{2}-0.05AB+0.02AD$ (1)

where A,B,C,D are the pyomelanin concentration, gold salt concentration, temperature and pH, respectively. The summary of the analysis of variance of model is presented in Table S2.

Table S2 Analysis of variance of fitted model used for nanoparticles synthesis. A, B, C, D are pyomelanin concentration, salt gold concentration, temperature and pH, respectively. A^2^, B^2^, C^2^, D^2^ represent quadratic coefficients while AB, AD, represent cross coefficients.

| **Source** | **Sum of squares** | **Mean of square** | **F value** | ***p*-value** |
| --- | --- | --- | --- | --- |
| Model | 12468.9 | 1246.8 | 19.29 | 0.000 |
| A | 3570.8 | 3570.8 | 55.25 | 0.000 |
| B | 954.1 | 954.1 | 14.76 | 0.001 |
| C | 1045.3 | 1045.3 | 16.17 | 0.001 |
| D | 972.0 | 972.0 | 15.04 | 0.008 |
| A^2^ | 700.2 | 700.2 | 10.83 | 0.001 |
| B^2^ | 2019.3 | 2019.3 | 31.24 | 0.000 |
| C^2^ | 2720.2 | 2720.0 | 42.09 | 0.000 |
| D^2^ | 1993.5 | 1993.5 | 30.84 | 0.000 |
| AB | 625.0 | 625.0 | 9.76 | 0.007 |
| AD | 1225.0 | 1225.0 | 18.95 | 0.000 |
| Lack of fit | 1225.4 | 73.2 | 16.90 | 0.057 |
| Error | 8.7 | 4.3 |  |  |
| R-squared | 92.34 |  |  |  |
| Adj-R-squared | 87.56 |  |  |  |
| Pred R-squared | 80.17 |  |  |  |

The F-test value for the model (i.e. 19.29) highlighted the significance of the model while that obtained for the lack of fit (i.e. 16.90) indicates that the model fits the experimental data and that the different parameters have significant effects on D50 value. The low *p-*value (p<0.05) of model terms, namely A, B, C, D, A^2^, B^2^, C^2^, D^2^, AB, AD, indicated that they affect significantly the D50 value. The correlation coefficient (R^2^), used to assess the fitness of the model, was calculated as 0.9234 which indicates that 92% of the variability in the response (i.e. D50 value) could be attributed to the independent variables (i.e. temperature, pH, HAuCl_4_, pyomelanin). Moreover, the high value of the adjusted determination coefficient (i.e. 0.8756) confirmed the high significance of the model ([1](#_ENREF_1)). The R-sq (pred) of 0.8017 is in agreement with the R-sq (adj) since their difference does not exceed 0.2 (for more details on data analysis refer to ([1](#_ENREF_1))). This highlighted a good correlation between the observed and predicted values.

***Model validation***

Graphical residuals analysis plays an important role on model diagnostic and validation ([2](#_ENREF_2)). Residuals are defined as the difference between experimental response and predicted response from the model. This analysis consists on scatters plot of normal probability of residuals distribution, residuals *vs* predicted plot and residuals *vs* experiment order.


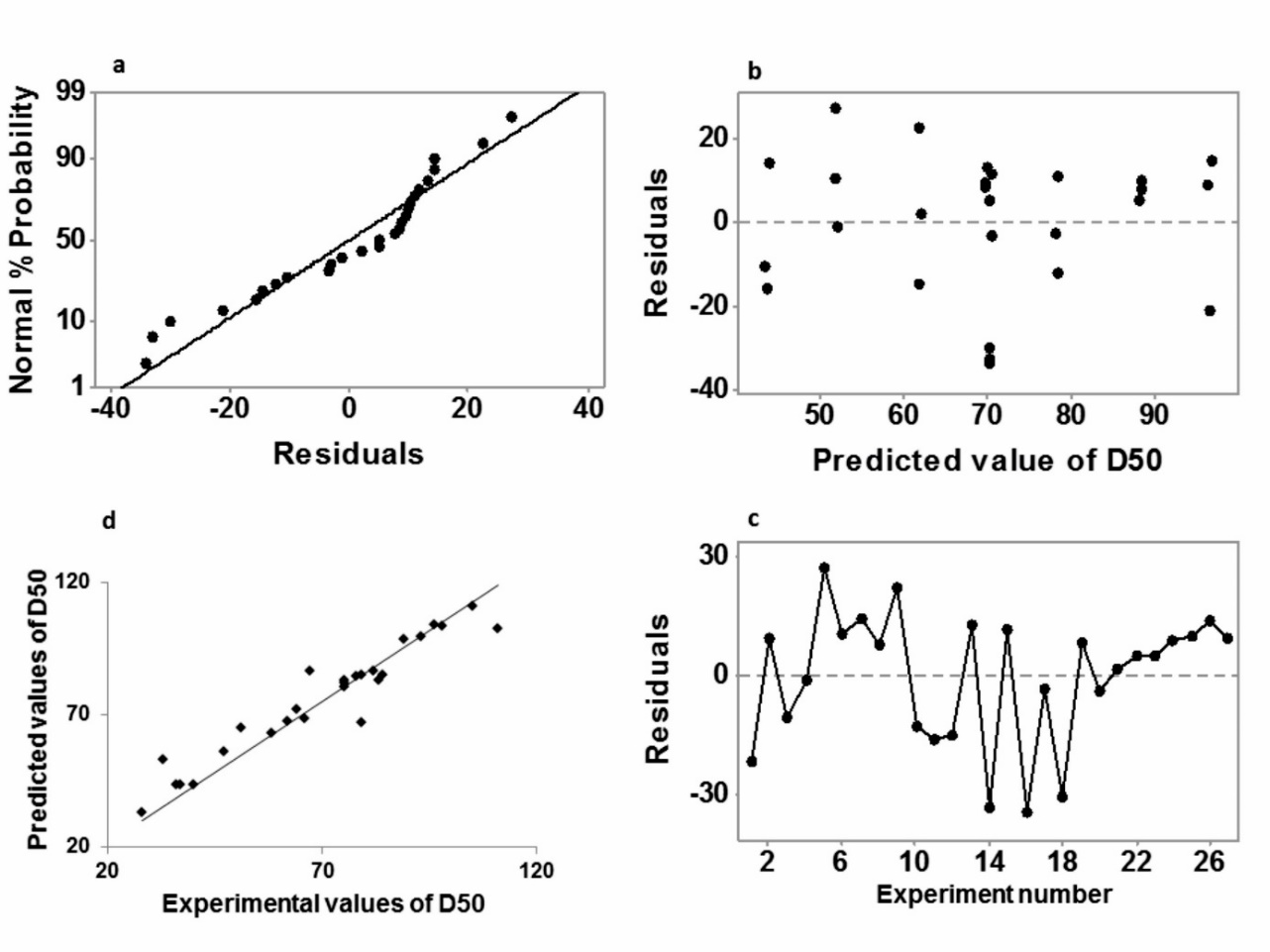


Figure S1 (a) Normal plots of residuals. (b) Residuals *vs* predicted values. (c) Residuals *vs* experiment number. (d) Predicted *vs* experimental values of AuNPs size.

The normal probability plot (Fig. S1a) provided a linear trend, which indicates that errors follow a normal distribution. A random scatter was also observed in the residuals *vs* predicted plot (Fig.S1b), and residuals appear homogeneously distributed in positive and negative interval. Moreover, the residuals for different PSD values obtained for each experimental condition (Fig S1c) appeared structureless and displayed a random pattern, which indicates that all residuals are not correlated with each other. Therefore, these residuals displays homogeneity, normality and independence and support the developed model. The Fig. S1d that represents the predicted *vs*. experimental values of AuNPs, highlighted a close relationship between these values indifferent experiments. This confirms the good predictive ability of the developed model.

As a model validation, three experiments were conducted, randomly, within the range of the experimental design (Table S3). The experimental D50 values were determined and compared with the predicted values. As shown in Table S3, the experimental value were closely related to the model calculated value, confirming thus the model validity.

**Table S3** Comparison between experimental and predicted values for nanoparticles size at different combinations of factors; D50 means median value.

| Experiment | Melanin (µg/mL) | Gold (mM) | pH | Temperature (°C) | D50 (nm) | |
| --- | --- | --- | --- | --- | --- | --- |
|  |  |  |  |  | Experimental | Predicted |
| 1 | 500 | 1.5 | 6.5 | 50 | 90 | 92 |
| 2 | 500 | 0.5 | 6 | 50 | 105 | 97 |
| 3 | 1000 | 0.5 | 6.5 | 50 | 49 | 52 |

1. Othman AM, Elsayed MA, Elshafei AM, Hassan MM. Application of response surface methodology to optimize the extracellular fungal mediated nanosilver green synthesis. J Genet Eng Biotechnol. 2017;15(2):497-504.

2. Chowdhury S, Yusof F, Faruck M, Sulaiman N. Process Optimization of Silver Nanoparticle Synthesis Using Response Surface Methodology2016.
